# Stability and change in potential correlates of physical activity and association with pubertal status among Norwegian children in the transition between childhood and adolescence

**DOI:** 10.1186/1479-5868-9-56

**Published:** 2012-05-11

**Authors:** Mekdes K Gebremariam, Ingunn H Bergh, Lene F Andersen, Yngvar Ommundsen, Mona Bjelland, Nanna Lien

**Affiliations:** 1 Department of Nutrition, Faculty of Medicine, University of Oslo, PO Box 1046, Blindern, N-0316, Oslo, Norway; 2 Department of Coaching and Psychology, Norwegian School of Sport Sciences, Oslo, Norway

**Keywords:** Adolescents, Physical activity, Correlates, Puberty, Tracking, Change

## Abstract

**Background:**

Whereas tracking and change in physical activity (PA) in children and adolescents have been well documented, studies investigating these patterns in its correlates are lacking. The present study aims to address this gap and in addition explore the impact of pubertal status on PA and its potential psychological and social-environmental correlates in a sample of Norwegian children over a 20-month period.

**Methods:**

A total of 885 students from 25 control schools of an intervention study, the HEalth In Adolescents (HEIA) study were included (mean age at baseline 11.2 (0.3)). The baseline took place in September 2007, the first follow-up in May 2008 and the second follow-up in May 2009. PA and its potential correlates (enjoyment of PA, self-efficacy related to barriers to PA, perceived support for PA from parents, friends and teachers, perceived social inclusion and perceived environmental opportunities for PA) were self-reported. Pubertal status was assessed using the Pubertal Development Scale. Repeated-measures ANOVA was used to explore changes. Tracking was assessed using Spearman’s rank order correlation. Pubertal groups were compared using ANOVA or ANCOVA (controlling for BMI). Multiple regression analyses were used to investigate whether pubertal stage at age 11 would predict levels of correlates and PA at age 13.

**Results:**

Potential correlates of PA and the behaviour itself were found to track moderately in the transition between childhood and adolescence. Small but significant changes in enjoyment of PA and teachers’ support for PA in both genders and in friends’ support for PA and perceived environmental opportunities for PA in females in a direction unfavourable to PA were detected. A few weak positive associations between pubertal stage and correlates of PA at age 11 were noted among boys.

**Conclusions:**

Enjoyment of PA, self-efficacy related to barriers to PA, perceived social support for PA, perceived social inclusion, perceived environmental opportunities for PA and the behaviour itself were found to be moderately stable in the transition between childhood and adolescence. Health promotion efforts in childhood targeting PA and its psychosocial and social-environmental correlates might have favourable effects in later years.

## Background

Regular physical activity (PA) provides wide-ranging short and long-term benefits for the health of children and adolescents. It helps in building and maintaining healthy bones and muscles; provides cardio-vascular benefits, enhances psychological health, and can help in the prevention of obesity [1-5]. In Norway, a significant increase in weight for height among children has been noted over the last 30 years [6]. Nevertheless, population surveys indicate that many children and adolescents do not meet requirements for PA [7]. Thus, interventions aimed at increasing PA in children and adolescents are of public health importance. Such interventions are based on the assumption that PA behavior tracks over time. Tracking refers to the tendency of individuals to maintain their rank or position within a group over time; its most common indicator is Spearman’s rank order correlation [8]. Several studies investigating tracking of PA in childhood and adolescence have been conducted. Two reviews summarized the results of these studies and indicated that tracking of PA in children and adolescents is weak to moderate [8,9].

Several psychosocial and social-environmental correlates of PA have been identified in the literature. Self-efficacy and social support are among the correlates most consistently associated with PA [4,7,10-12], also found previously in the HEIA study [13]. Other correlates of PA described in the literature include enjoyment of PA and perceived environmental opportunities for PA [7,12,14,15]. Studies investigating tracking of these correlates of PA are lacking. The Social Cognitive Theory suggests that the relationship between determinants and behavior is reciprocal [16]. Changes in determinants are thus likely to co-vary with changes in PA [17]. This suggestion has been further supported by studies which documented that psychosocial variables at baseline (e.g. self-efficacy) were poorer correlates of PA change than changes in these psychosocial correlates [17,18]. As PA appears to track over time, one would thus also assume that some of its psychological and social-environmental correlates would display that pattern, although stability in PA behavior might also be related to other factors such as the behavior becoming habitual or hereditary disposition [8]. Investigating the tracking of psychological and social-environmental correlates is important as it can indicate the proper timing of interventions targeting these correlates. Since levels of PA are known to decrease in childhood and adolescence [10,19], and are expected to co-vary with and be predicted by correlates such as enjoyment, self-efficacy, perceived social support and perceived environmental opportunities for PA, investigating the patterns of change in these correlates over time is also important.

The transition into adolescence is a critical age period as dynamic physical, psychosocial and behavioral changes are expected to occur [20]. Each of these changes might be associated with pubertal maturation. Puberty and in particular the timing of pubertal maturation relative to one’s peers might be associated with levels of PA [9,20]. Maturation is also believed to influence the tracking of PA in this period [8,9]. Findings of a recent study suggest that in females and in early adolescence, tracking of PA can be improved if differences in the timing of maturation are controlled for [21]. A need to better understand the impact of maturation on PA behaviors and beliefs has been stressed [20,22].

The main aim of this paper is to explore stability and change of enjoyment of PA, self-efficacy related to barriers to PA, perceived parental support for PA, perceived support from friends for PA, perceived support from parents for PA, perceived environmental opportunities for PA and social inclusion over a 20-month period among Norwegian children in the transition between childhood and adolescence. Stability and change of self-reported PA will also be described, in order to indicate how these relate to stability and change of correlates. The study will in addition assess the impact of pubertal timing on PA and its correlates.

## Methods

### Design and sample

The participants in this study are students from 25 control schools of the HEalth In Adolescents (HEIA) study. The overall aim of the HEIA study was to develop and evaluate a multi-component intervention study aimed at healthy weight development through diet and PA [23]. A total of 177 schools were invited, and 37 schools accepted the invitation. Schools were included in this study if they had a minimum of 40 enrolled pupils in the 6^th^ grade. Schools were thus recruited from the largest towns/municipalities in seven counties from the Eastern part of Norway. Twenty five of the schools were randomly assigned to the control group and twelve to the intervention group.

All 6^th^ graders in the 25 control schools included in this study and their parents/legal guardians were invited to participate in the baseline (BL) study which took place in September 2007. Parental consent was obtained for 1014 children (response rate 73 %) from these schools. A total of 975 students (96 % of the 1014 returning parental informed consent) participated at baseline (BL). The first follow-up (T1) study took place in May 2008, and 934 (96 %) of those participating at BL participated. The second follow-up (T2) was conducted in May 2009, and 885 (95 %) of those participating at BL and T1 participated.

Ethical clearance was obtained from the Regional Committees for Medical Research and the Norwegian Social Science Data Service.

### Data collection

Information about PA and its correlates was self-reported. During school hours, the children answered an internet-based questionnaire over a period of approximately 45 minutes. Two research team members provided the children with technical assistance, answered questions they had and ensured that they replied independently from each other.

### Measures

#### Potential correlates of PA

Two psychological correlates, enjoyment of PA [24], and self-efficacy related to barriers for PA, adapted and modified from Motl et al. 2006 [25] and Lytle 2009 [26], were assessed by 5-item scales.

Five social-environmental correlates were included: perceived social support from friends, assessed by a 3-item scale, perceived social support from parents, assessed by a 5-item scale [27], perceived social support from teachers, assessed by a 3-item scale [28]. Perceived environmental opportunities to be physically active were assessed by a 4-item scale [27]; perceived social inclusion related to the school and class environment was assessed by a 6-item scale based on a “social capital measure” [29]. Evidence of validity of these scales has been documented [6,29-31].

The answer categories related to social support were phrased “almost never or never”, “1 to 2 times a week”, “3 to 4 times a week”, “almost every day” and “every day”. The other correlates were measured using scales in which the items were rated on a 5-point Likert-type scale coded 1 (lowest) to 5 (highest) and phrased “totally disagree” to “totally agree” with a neutral midpoint.

Cronbach’s alpha ranged from 0.64 to 0.85 at all three time points for all the correlates. For enjoyment of PA, one item had to be deleted when the composite score was computed, so as to obtain an acceptable Cronbach’s alpha value (≥ 0.6). Adequate test-retest correlations were found from a separate test-retest study conducted among 111 6^th^ graders 10–14 days apart before the first data collection (Table 1). Support from friends, environmental opportunities and social inclusion were measured only at BL and T2.

**Table 1:** Potential psychosocial and social-environmental correlates of physical activity (PA) among Norwegian 11–13 year-old children

		**Mean(SD)**			**α**^a^	**r**^b^
		**Baseline**	**T1**	**T2**		
Enjoyment of PA (4 items)	Girls^c^	4.13(0.72)	4.10(0.70)	3.96(0.75)	0.71,0.70,0.73	0.70
	Boys^c^	4.12(0.78)	4.05(0.79)	3.94(0.88)		
Self-efficacy related to barriers to PA (5 items)	Girls	3.89 (0.73)	3.96(0.68)	3.87 (0.76)	0.75,0.73,0.77	0.68
	Boys	3.83 (0.79)	3.90(0.81)	3.85 (0.86)		
Perceived parental support (5 items)	Girls	2.34 (0.76)	2.42(0.78)	2.24 (0.75)	0.66,0.68,0.69	0.70
	Boys	2.40 (0.76)	2.46(0.82)	2.37 (0.85)		
Perceived support from teachers (3 items)^d^	Girls^c^	1.68(0.65)	1.61(0.65)	1.40(0.52)	0.71,0.67,0.70	0.59
	Boys^c^	1.68(0.74)	1.61(0.65)	1.52(0.66)		
Perceived support from friends (3 items)^d,e^	Girls^c^	2.85(0.98)	NA^f^	2.60(0.92)	0.83, 0.85	0.76
	Boys	3.06 (1.01)		2.98 (1.01)		
Perceived environmental opportunities (4 items)	Girls^c^	4.26(0.72)	NA^f^	4.07(0.84)	0.66, 0.72	0.65
	Boys	4.25 (0.79)		4.14 (0.92)		
Perceived social inclusion	Girls	4.40 (0.60)	NA^f^	4.34 (0.66)	0.81, 0.83	NI^g^
(6 items)	Boys	4.45 (0.61)		4.37 (0.70)		

^a^ Chronbach’s alpha at BL, T1 and T2.

^b^ Test-retest correlation coefficient computed from separate test-retest study 10–14 days apart (n = 111). All coefficients significant at the 0.01 level.

^c^ Statistically significant change between BL and 2 year follow-up (p < 0.01).

^d^Statistically significant difference between boys and girls at T2(p < 0.01).

^e^Statistically significant difference between boys and girls at BL (p < 0.01).

^f^ Not assessed at T1.

^g^ NI: measure not included in test-retest.

N = 466 for boys and 419 for girls and varies slightly between variables.

### Physical activity

The children were asked to report the time they spend on PA: “For how long do you think you are physically active (walk, bicycle, work out, play, and engage in sports) during the course of a normal day?” The answer categories were: less than 15 mins, 15–29 mins, 30–59 mins, 1–1.5 hr, 1.5-2 hrs, more than 2 hrs. The question was adopted and modified from Pro Children [32]. A moderate test-retest correlation coefficient (0.68) was obtained from the test-retest study. Recoding into minutes per day was done using mid-points.

### Pubertal status

The Pubertal Development Scale (PDS) is based on the pubertal category scores defined by Carskadon and Acebo [33]. PDS for boys included body hair growth, voice change, and facial hair. For girls, PDS included body hair growth, breast development, and menarche. The students self-reported this information. Test-retest correlations for these items ranged from 0.38 - 0.90 [23]. The categories were: pre-pubertal, early pubertal, mid-pubertal, late pubertal or post-pubertal. The last three categories were merged for the analyses, due to a low number of male children in the last categories.

BMI was calculated as weight/height^2^. Weight and height were objectively measured by same sex project staff [23].

### Statistical analysis

Since schools were the unit of measurement in this study, we checked for clustering effect through the Linear Mixed Model procedure. Only 0.2 - 3.6 % of the unexplained variance in the variables investigated was at the school level. Adjustment for clustering effect was therefore not done.

Data was stratified by gender. Mean values (standard deviations) were calculated. Repeated-measures ANOVA was used to assess changes in the correlates and to assess changes in PA between T1 and T2 which represented similar seasons. Effect sizes (partial eta squared) were used to assess the magnitude of these changes. The cut-offs suggested by Cohen were used for interpretation of effect sizes (1 % = small effect, 6 % = moderate effect, 14 % = large effect) [34].

Tracking (stability) was assessed using Spearman’s rank order correlation coefficient. Correlation coefficients of <0.30 indicate low correlation, 0.30 to 0.60 moderate, and > 0.60 moderately high correlation [9]. Correction for attenuation of the coefficients, that is correction for measurement errors, was done using test-retest measures [35]. Test-retest reliability was assumed to be the same at BL, T1 and T2.

Differences in correlates across pubertal groups were assessed using ANOVA with post-hoc tests (Bonferroni adjustment). For PA, ANCOVA was used in order to adjust for BMI. Assessment of whether timing of pubertal maturation at age 11 would predict level of correlates at age 13 was done using multiple regression analysis, after controlling for baseline values. The same analysis was conducted for PA, controlling for baseline values and for BMI at baseline. Comparisons between genders in levels of PA and correlates were conducted using independent samples t-tests.

Attrition analysis was performed using independent samples *t*-test and chi-squared test of proportions to determine differences in baseline characteristics between participants and drop-outs (n = 90).

Due to the large number of analyses performed, Bonferroni correction was done and the p-value was set to 0.01.

All statistical analyses were performed by IBM® SPSS® Statistics, version 18.0 (IBM Corp., Somers, New York, USA).

## Results

### Descriptives, stability and change

A total of 885 students (47 % girls and 53 % boys) who participated at all three time points were included in the analyses. The mean age at baseline was 11.2 (0.3) yrs. In girls, 10.7 % were pre-pubertal, 19.4 % early pubertal, 69.9 % were mid/late/post-pubertal at baseline. The respective proportions in boys were 32.4 %, 51.8 % and 15.8 %. BMI at BL was 18.0 (2.7) kg/m^2^ in girls and 17.9 (2.7) kg/m^2^ in boys.

The attrition analysis showed that there was no significant difference in demographic and outcome characteristics at baseline between participants and drop-outs (data not shown).

The mean values for the different psychological and social-environmental correlates at BL, T1 and T2 are presented in Table 1. The lowest mean values for both boys and girls were obtained for the social support variables and in particular for perceived support from teachers. The highest values were obtained for enjoyment of PA, perceived social inclusion and perceived environmental opportunities. The independent samples t-tests showed small but significant differences between boys and girls in perceived social support from friends at BL (p = 0.002) and T2 (p < 0.001), with boys perceiving more social support for PA from friends. At T2, there was also a significant difference in perceived social support for PA from teachers (p = 0.004), girls perceiving less social support from teachers.

Among girls, repeated-measures ANOVA tests showed small but significant decreases (p < 0.001) in enjoyment of PA, perceived support from friends, perceived support from teachers and perceived environmental opportunities between BL and T2. Among boys, there were small but significant decreases (p < 0.001) in enjoyment of PA and perceived support from teachers. The effect sizes based on partial eta squared statistic were small-to-moderate except for perceived support from teachers for females for which the effect size was large (data not shown). The mean levels of PA are presented in Table 2. When similar seasons were compared, PA declined slightly in girls but remained stable among boys within a one-year period.

**Table 2:** Total physical activity at baseline, eight and twenty months follow-up among Norwegian children

	**Girls (419)**		**Boys (466)**	
	Mean^a^	SD	Mean^a^	SD
Baseline	81.81	34.24	87.33	33.43
T1	95.08^b^	30.12	97.54	29.33
T2	90.10^b^	31.68	95.38	30.11

^a^ In minutes per day.

^b^ Statistically significant difference between T1 and T2 (p < 0.01).

Baseline was in the Spring season, time 1 and 2 were in the Fall season.

Spearman’s rank order correlation coefficients showed the presence of significant moderate tracking of correlates of PA in both genders (Table 3). The coefficients corrected for measurement error were moderate-to-moderately large. There was no significant difference between correlation coefficients adjusted for pubertal status and unadjusted coefficients. Therefore unadjusted coefficients are reported (Table 3). For girls, the coefficients ranged from 0.30 for perceived environmental opportunities to 0.50 for perceived parental support for girls over the 20-month period. For boys, the coefficient ranged from 0.33 for perceived support from teachers to 0.52 for self-efficacy. Coefficients corrected for attenuation ranged from 0.46 to 0.71 in girls and 0.56 - 0.76 in boys over the 20-month period. Higher coefficients were obtained for adjacent time points.

**Table 3:** Tracking coefficients over 20 months for potential psychosocial and social-environmental correlates of physical activity

		**Girls (419**^a^**)**				**Boys (466**^a^**)**			
		**r**		**r'**		**r**		**r'**	
		**T1**	**T2**	**T1**	**T2**	**T1**	**T2**	**T1**	**T2**
Enjoyment of PA	BL	0.52	0.37	0.74	0.53	0.55	0.45	0.78	0.64
	T1	1	0.45	1	0.64	1	0.52	1	0.74
Self-efficacy	BL	0.55	0.45	0.81	0.66	0.56	0.52	0.82	0.76
	T1	1	0.49	1	0.72	1	0.53	1	0.78
Perceived parental	BL	0.54	0.50	0.77	0.71	0.51	0.47	0.73	0.67
support	T1	1	0.57	1	0.81	1	0.49	1	0.70
Perceived support from	BL	0.37	0.30	0.63	0.51	0.34	0.33	0.58	0.56
teachers	T1	1	0.35	1	0.59	1	0.38	1	0.64
Perceived support from	BL	NA	0.36	NA	0.47	NA	0.43	NA	0.56
friends	T1	1	NA	1	NA	1	NA	1	NA
Perceived environmental	BL	NA	0.30	NA	0.46	NA	0.42	NA	0.65
opportunities	T1	1	NA	1	NA	1	NA	1	NA
Perceived social inclusion^b^	BL	NA	0.37			NA	0.40		
	T1	1	NA			1	NA		

All Spearman’s correlation coefficients are significant at 0.01 level.

r’ = coefficient corrected for attenuation using test-retest values. Test-retest was assumed to be the same at the three time points.

NA = not assessed at T1.

^a^N varies slightly between variables.

^b^ measure not included in test-retest, therefore r’ not computed.

Tracking coefficients of PA (and coefficients corrected for attenuation) between the first and second follow-up which represented similar seasons were 0.43 (0.63) for girls and 0.47 (0.69) for boys.

### Impact of pubertal status

Small but significant differences in mean values of some correlates at BL between pubertal development groups were detected among boys (Table 4). Post-hoc tests showed that boys in the mid/late/post-pubertal stage experienced higher enjoyment of PA (p = 0.002), higher support from teachers (p < 0.001) and higher support from parents (p = 0.001) than boys in the pre-pubertal stage. Boys in the mid/late/post-pubertal stage also perceived higher support from parents (p = 0.007) and higher support from teachers (p = 0.001) than those in the early pubertal stage. The associations were nevertheless weak. Pubertal status at BL was not associated with PA at BL and did not predict levels of PA and correlates at age 13 in either of the genders (data not shown).

**Table 4:** Physical activity and its correlates, association with pubertal stage at age 11 among Norwegian children

	**Girls (402**^a^**)**			**P**^b^	**Boys (423**^a^**)**			**P**^b^
	**Pre-pubertal (11 %)**	**Early pubertal (19 %)**	**Mid/late/post pubertal (70 %)**		**Pre-pubertal (32 %)**	**Early pubertal (52 %)**	**Mid/late/post pubertal (16 %)**	
Self-reported PA (mins/day)^c^	78.6	78.8	83.9	0.38	86.8	88.0	94.0	0.31
Enjoyment of PA	4.19	4.14	4.13	0.92	3.99	4.16	4.39	0.003
Self-efficacy	3.88	3.91	3.91	0.96	3.77	3.86	4.04	0.06
Perceived parental support	2.23	2.3	2.36	0.47	2.32	2.38	2.70	0.003
Perceived support from teachers	1.59	1.69	1.69	0.65	1.60	1.63	2.02	<0.001
Perceived support from friends	2.87	2.73	2.88	0.49	2.98	3.08	3.22	0.26
Perceived environmental opportunities	4.21	4.20	4.321	0.28	4.27	4.22	4.36	0.42
Perceived social inclusion	4.55	4.39	4.41	0.32	4.46	4.45	4.56	0.39

^a^ N varies slightly between variables.

^b^P values for the difference between pubertal groups computed using ANOVA except for ^C^ for which ANCOVA was used (adjustment for BMI done).

## Discussion

Results of this study showed that self-reported PA tracked moderately between the ages of 12 and 13. Previous reviews have indicated low to moderate tracking of PA in childhood and adolescence [8,9], although comparison between studies is complicated among other things by the different lengths of follow-up. Adjusted coefficients were found to be clearly higher than unadjusted ones [8], as was found in this study. However, adjustment was done in very few previous studies. Tracking of PA in children and adolescents has been well discussed in the literature, and will therefore not be repeated here. The documented decline in PA among girls is also in accordance with findings in children in this age group [19]. The decline noted in this study was nevertheless very small. A Norwegian study with a large population-based sample has shown that PA levels were higher in the Spring compared to the Fall season among 9 year-olds [36]. What appears to be an increase in PA between BL and T1 is thus most probably related to these seasonal variations.

Despite the long known tracking of PA behaviour, investigation of the stability of factors associated with it has been lacking. PA is influenced by different factors including genetic predisposition as well as psychological and social-environmental factors [4,8,10-12,37]. From the standpoint of public health promotion, particular attention to factors amenable to change should be given. This study shows that potential psychological and social-environmental correlates of PA appear to be well established at a young age, with moderate to moderately-high tracking. Higher tracking coefficients in both genders were found for self-efficacy and parental support, both robust correlates of PA. Low to moderate tracking of psychological and social-environmental correlates of PA was reported in a study of young adults and over a 7 year period [16]. However, no study of tracking of correlates of PA in this age group could be identified.

Small but significant changes in enjoyment of PA and teachers’ support in both genders and in friends’ support and perceived environmental opportunities in females in a direction unfavourable to PA were detected. The changes documented in this study are rather small and probably not of much practical significance in the studied period. Nevertheless, they might be indicative of a trend which is getting established. Unless addressed, these changes might increase further and adversely impact PA and might get exacerbated when the children go through school transition at the end of 7^th^ grade, as indicated in a previous study [22].

In light of the moderate tracking of potential correlates of PA together with the unfavorable changes noted, early health promotion efforts to make cognitions, beliefs and perceptions related to PA more positive would seem important. Such efforts can include the enhancement of social support for PA, as well as getting children to begin exercising and providing them with feedbacks, which is believed to be critical in the enhancement of self-efficacy for PA [12]. Tracking is a positive phenomenon for children with high levels of PA and with positive cognitions, beliefs and perceptions related to PA. Health promotion efforts should thus also be aimed at such children in order to help them maintain these levels.

Finally, no effect of pubertal timing on PA at BL and 20 months later was noted. Sherar et al. 2010 summarized the results of the few existing studies looking at the impact of biological maturity on PA [20]. They found inconsistent results for both genders. Associations, when noted, were generally low, as was also found in this study. They concluded that differences may stem from different factors, including the methods used to assess both maturity and PA. Evidence of validity and reliability of the method used to assess pubertal stage in this study has been documented [33]. However, the measure gives a rough assessment of pubertal status and has weaknesses compared to more direct measures, such as the possibility of social desirability bias related to the self-report. PA was also self-reported. However, the differences between studies might also reflect contextual differences since pubertal maturation has important societal and cultural dimensions [20], which might in turn determine whether or not it will influence PA. The absence of an association between the timing of pubertal maturation and PA beliefs and perceptions at age 11 among females, and the few weak associations among males should also be interpreted in light of this. Whereas early maturation in girls is hypothesized to have many potential negative psychosocial impacts, including less enjoyment of PA as well as less social support from parents and peers [38], findings of this study do not support those hypotheses, at least with regards to how these are perceived by children. Corresponding literature for boys is limited; but early maturation in boys is believed to have advantages, because their body changes conform to the socio-cultural ideal [39]. In addition, early maturation and advanced physical development might lead to a better athletic ability [39] and thus early maturers can more easily be positively reinforced for their sport and physical activity achievements. These facts might at least in part explain the slightly higher enjoyment of PA and perceived support for PA from parents and teachers in early maturing boys in this study. Worth emphasizing is that results might have been different if comparison between extreme maturity groups was conducted, which was impossible in this study because of low numbers of children in these groups, due to the age at which pubertal status was assessed. A progress to higher maturity stages when pubertal changes become more visible is likely to be related to more personal and social influences on the children.

### Strengths and weaknesses

The study has both weaknesses and strengths. Self-reported measures in children present problems of validity and reliability. Nevertheless, good test-retest reliability was obtained for the measures used. Good and stable estimates of internal consistency reliability of the instruments used to measure the correlates were also found across all the time points. In their review of studies of tracking of PA, Telama et al. 2009 indicated that many studies did not account for low reliability of measures used and suggested the use of reliability information in order to correct correlations for measurement errors [8], which was done in this study.

One methodological consideration is that the children might give more accurate answers after being previously exposed to the same instrument, and also with increasing age, although it is difficult to estimate the magnitude of such effects. This can influence the tracking coefficients, and the changes noted.

The question assessing PA was general and intended to provide a measure of total PA. It was mainly used to look at trends over time in this study, and the use of self-reported measures of PA for such purposes appears justified [40]. In addition, studies have shown that simple, overall self-report PA questions correlated significantly with other measures, and have relatively satisfactory validity [41]. However, such a measure is unlikely to capture sporadic activity, as an accelerometer would have. As there are known seasonal variations in PA among children in Norway [36], stability and change in PA were assessed only between T1 and T2 which represented similar seasons. Tracking is influenced by the duration of follow-up. The follow-up period in this study is relatively short. Nevertheless, the transition into adolescence is a period during which rapid changes are expected to occur. It is also a critical period from the perspective of physical education and lifestyle development [8]. The study is also limited to a single geographical area making generalizability limited.

The study provides new insights into the tracking and change of potential correlates of PA in young children, and is, to our knowledge, the only study addressing this issue in this age group. The sample size for the study was large and the rate of retention was also high.

## Conclusion

Enjoyment of PA, self-efficacy related to barriers to PA, social support for PA, perceived social inclusion, perceived environmental opportunities for PA and the behaviour itself are moderately stable in the transition between childhood and adolescence. Small but significant decreases in enjoyment of PA and teachers’ support for PA in both genders and in friends’ support for PA, perceived environmental opportunities for PA and self-reported PA in females were detected. Few weak positive associations between pubertal stage and correlates of PA at age 11 were noted among boys.

The moderate stability of PA and its potential psychosocial and social-environmental correlates indicates that health promotion efforts in childhood targeting PA and these potential correlates will have favourable effects in later years.

Studies following children as they progress into school transition are needed to further explore the documented patterns of stability and change.

## Abbreviations

BL, Baseline; BMI, Body mass index; HEIA, HEalth In Adolescents; PA, Physical activity; PDS, Pubertal Development Scale; T1, First follow-up; T2, Second follow-up.

## Competing interests

The authors declare they have no competing interests.

## Authors’ contributions

M.K.G. conducted the statistical analyses and wrote the first draft of the manuscript and made the greatest contribution to the paper. N.L., I.H.B, L.F., M.B. and YO participated in designing the study, project planning and data collection. All authors have critically revised the manuscript, and read and approved the final version of the manuscript.
